# γ-Al_2_O_3_ supported Pd@CeO_2_ core@shell nanospheres: salting-out assisted growth and self-assembly, and their catalytic performance in CO oxidation†
†Electronic supplementary information (ESI) available: XPS, BET analyses of the as-obtained Pd@CeO_2_ core@shell nanospheres; TEM images of other control experiments, including using KCl and KI instead of KBr to synthesize Pd–CeO_2_ system; using KCl and KI to induce the forming of Au@CeO_2_ and Pt@CeO_2_, respectively. See DOI: 10.1039/c4sc03854a


**DOI:** 10.1039/c4sc03854a

**Published:** 2015-02-27

**Authors:** Xiao Wang, Dapeng Liu, Junqi Li, Jiangman Zhen, Fan Wang, Hongjie Zhang

**Affiliations:** a State Key Laboratory of Rare Earth Resource Utilization , Changchun Institute of Applied Chemistry , Chinese Academy of Sciences , Changchun 130022 , Jilin , China . Email: liudp@ciac.ac.cn ; Email: hongjie@ciac.ac.cn; b Graduate School of The Chinese Academy of Sciences , Beijing 100039 , China

## Abstract

In this paper, we have successfully demonstrated the clean synthesis of high-quality Pd@CeO_2_ core@shell nanospheres with tunable Pd core sizes in water, and furthermore loaded the as-obtained Pd@CeO_2_ products on commercial γ-Al_2_O_3_*via* electrostatic interaction. KBr here plays two key roles in inducing the growth and self-assembly of Pd@CeO_2_ core@shell nanospheres. First, Br^–^ ions can retard the reduction of Pd^2+^ ions *via* the formation of the more stable complex of [PdBr_4_]^2–^ so as to tune the size of Pd cores. Second, it greatly decreases the colloidal stability, and hence the surface polarity-weakened Pd and CeO_2_ NPs have to spontaneously self-assemble into more stable and ordered structures. Among different-sized Pd samples, the as-obtained 8 nm-Pd@CeO_2_/Al_2_O_3_ one exhibits the best performance in catalytic CO oxidation, which can catalyze 100% CO conversion into CO_2_ at 95 °C, which is much lower than the previously reported CeO_2_-encapsulated Pd samples.

## Abstract 2

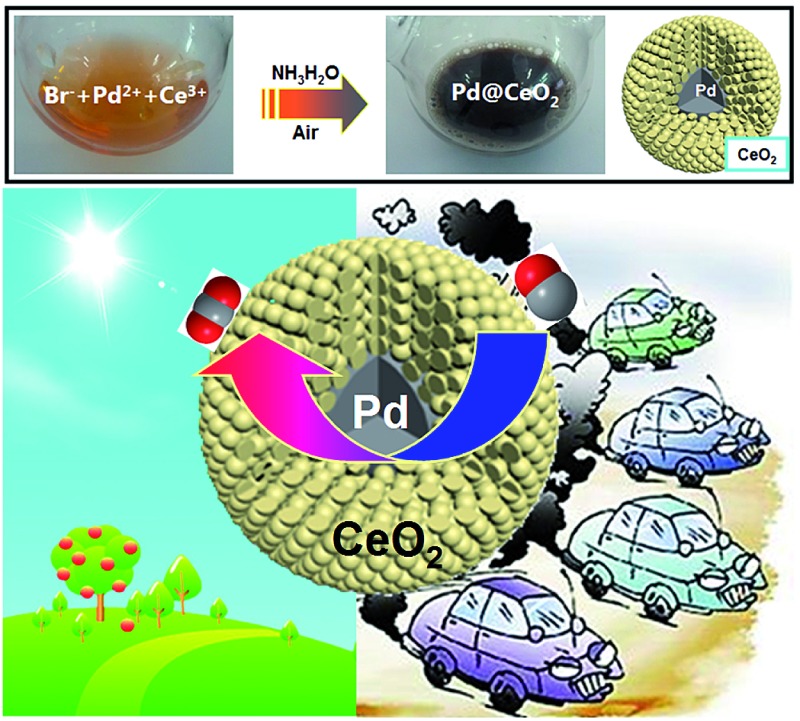
Highly active Pd@CeO_2_ core@shell nanospheres with tunable Pd core sizes for catalytic CO oxidation.

## 1. Introduction

Pd based catalysts have received continuous attention for decades in a wide range of applications.^1^–^5^ For example, in organic chemistry, Pd(0) or its compounds have become indispensable to a mass of carbon–carbon bond forming reactions like Suzuki, Heck, and Stille coupling.^6^–^10^ Besides, supported PdO_*x*_ has been recognized as one of the best catalysts for catalytic CH_4_ oxidation.^11^ Due to the harsh conditions they often work in, especially under high temperatures, the Pd catalysts find it hard to resist aggregation or growth, resulting in the heavy loss of catalytic active centers and hence severe catalytic degradation and even inactivation. That means it is very important for Pd NPs to well keep their original sizes and shapes and thus their long-lasting optimal catalytic performance. Thus, how to improve the thermal stability of Pd nanoparticles (NPs) becomes a serious and urgent global problem that needs to be solved. Among different solutions, to form core@shell nanostructures has been identified as the most efficient way.^12^–^18^ By densely coating with shell components, the Pd core can be physically protected, the mass transformation of which is able to be prevented during both the synthesis and the long-term catalytic cycling.

A variety of Pd@metal oxide core@shell nanostructures have been successfully fabricated, including Pd@SiO_2_,^19^,^20^ Pd@ZnO,^21^ Pd@TiO_2_,^22^ and Pd@CeO_2_.^23^ Among these oxides, CeO_2_ seems more distinctive as the shell because of its excellent chemical and physical properties such as extra-low-temperature oxygen storage capacity^24^ and the ability to generate strong synergistic effects associated with noble metals.^25^–^28^ Recently, Pd@CeO_2_ core@shell nanostructures have been successfully obtained *via* self-assembly that occurs between functionalized Pd NPs and Ce(iv) alkoxides in organic solutions.^29^–^32^ However, there still exist two problems in the Pd@CeO_2_ system. Firstly, such multi-step assembly requires precise control and complex surface modification, and moreover the unavoidable usage of expensive and toxic organic surfactants and solvents that are harmful for our health and environment. Secondly, the use of supports gives important benefits for enhancing the thermal stability of CeO_2_. However, the hydrophobic alkyl capping of such Pd@CeO_2_ core@shell nanostructures is repelled by the hydrophilic surface of metal oxide supports, especially the most used high-surface-area γ-Al_2_O_3_ powders. Before loading Pd@CeO_2_, it is inevitable to change the polarity of the γ-Al_2_O_3_ supports *via* complex and expensive surface modification.^35^ It is not conducive to large-scale synthesis and has seriously limited applications of catalysts. So in this study, the following two points have been focused upon in order to address the above mentioned issues. One is the aqueous clean synthesis of high-quality size-tunable Pd@CeO_2_ core@shell nanostructures without usage of organics, and the other is the direct assembly of them on γ-Al_2_O_3_ supports uniformly without any surface pretreatment.

Recently, an auto-redox relationship that happens between Ce^3+^ and noble metal ions has aroused scientists' interests. Following this strategy, Ag@CeO_2_ core@shell^33^–^35^ and Pt@CeO_2_ multi-core@shell nanospheres^36^ have been facilely obtained, and moreover the whole preparation processes are totally clean without using any expensive and toxic organopalladium compounds, long-chain alkyl surfactants and high boiling solvents, as well as complex synthetic steps and laborious post-treatment. More importantly, the naked surface of the Ag@CeO_2_ and Pt@CeO_2_ nanospheres favor them exhibiting better catalytic performance compared with those modified by surfactants. However unfortunately, such an advanced strategy does still not work in the very important Pd@CeO_2_ system (see Fig. S1 in ESI†) due to lack of enough driving force. Despite thus obtained bare Pd NPs are only supported on CeO_2_ with no ordered hybrid structure, it is still highly expected to find an appropriate thermodynamic condition to realize the desirable Pd@CeO_2_ core@shell ones.

**Scheme 1:**
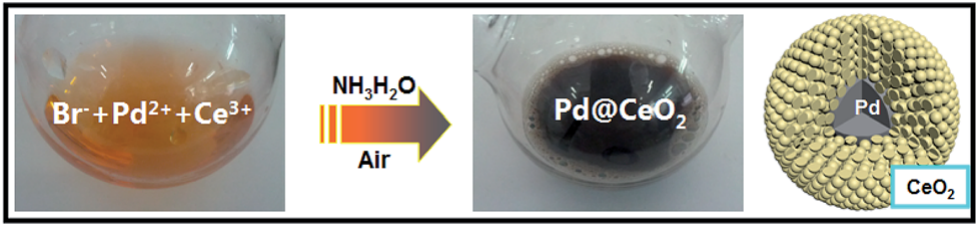
The synthetic process of Pd@CeO_2_ core@shell nanospheres.

Herein, the classic “salting-out effect” was first proposed in the clean synthesis of core@shell nanostructures. The salting-out effect can be generally explained as introducing a large number of inorganic salts into the mother solution so as to make the original solute supersaturated and thus being separated by crystallization, mostly applied in the biological areas for extraction. Until now, from a view point of synthesis, the most successful strategy of noble metal-based core@shell nanostructures is the seeded growth process. Tang and co-workers have reported a series of works on such an area.^37^–^39^ However, after carefully checking out the previous reports, there is still no report on such synthesis of NPs. In this work, we have tried to utilize the salting-out strategy to disclose the evolution of how high-quality Pd@CeO_2_ core@shell nanospheres were formed, and how inorganic metal salts (KCl, KBr or KI) worked during the growth and the self-assembly process of Pd and CeO_2_ components. Furthermore, we have systematically studied the size and the support (γ-Al_2_O_3_) influences on the catalytic performance of the Pd@CeO_2_ core@shell nanospheres towards CO oxidation. The whole synthesis is shown in Scheme 1.

## 2. Experimental section

### 2.1 Synthesis of 13 nm Pd@CeO_2_ core@shell nanospheres

The whole synthetic process is described in Scheme 1. 300 mg of KBr was dissolved in 20 mL of H_2_O first. The solution was heated to 60 °C for 10 min. Then 0.8 mL of Na_2_PdCl_4_ aqueous solution (1 mmol of PdCl_2_ and 2 mmol of NaCl were dissolved in 16 mL of H_2_O) and 2 mL of Ce(NO_3_)_3_ aqueous solution (0.1 M) were added followed by dropping 5 mL of ammonia (50 μL 25% ammonia dissolved in 20 mL of H_2_O). The mixture was heated at 60 °C for 1 h. After cooling down to room temperature, the product was purified by centrifugation and washed with water three times.

Pd@CeO_2_ core@shell nanospheres with other Pd core sizes were synthesized under similar reaction conditions, just by changing the amount of KBr and ammonia. For example, 300 mg KBr and 200 μL 25% ammonia, 150 mg KBr and 50 μL 25% ammonia, and 500 mg KBr and 50 μL 25% ammonia were used to synthesize 8 nm, 11 nm, and 17 nm sized Pd@CeO_2_ core@shell nanospheres, respectively. The final samples are named as 8 nm-, 11 nm-, 13 nm-, and 17 nm-Pd@CeO_2_, according to the size of Pd NPs.

### 2.2 Synthesis of Pt@CeO_2_ multi-core@shell nanospheres

The synthesis was similar to the above process. A certain amount of KI was dissolved in 20 mL of H_2_O first. The solution was heated to 60 °C for 10 min. Then 0.8 mL of K_2_PtCl_4_ aqueous solution (1 mmol of K_2_PtCl_4_ dissolved in 16 mL of H_2_O) and 2 mL of Ce(NO_3_)_3_ aqueous solution (0.1 M) were added, followed by dropping 5 mL of diluted ammonia (50 μL 25% ammonia dissolved in 5 mL of H_2_O). The mixture was heated at 60 °C for 1 h. After cooling down to room temperature, the product was purified by centrifugation.

### 2.3 Synthesis of Au@CeO_2_ core@shell nanospheres

The synthesis was similar to the above process, except using HAuCl_4_ and KCl instead of Na_2_PdCl_4_ and KBr, respectively.

### 2.4 Synthesis of CeO_2_ NPs

The synthesis was the same as the synthesis of Pd@CeO_2_ except for adding Na_2_PdCl_4_.

### 2.5 Pd@CeO_2_ supported on γ-Al_2_O_3_

100 mg commercial γ-Al_2_O_3_ powder was mixed with 100 mL water and 100 μL 25% ammonia. The mixture was heated at 95 °C for 2 h. Meanwhile, 100 mg of the Pd@CeO_2_ sample was dispersed in 50 mL water by ultrasound treatment. Finally, the as-obtained Pd@CeO_2_ colloids were dropped into the γ-Al_2_O_3_ dispersion very slowly. After heated at 95 °C for another 2 h, the products could be collected by centrifugation.

### 2.6 Heat treatment

All the samples have been calcined before catalytic tests. The calcination processes were performed in air at a heating rate of 5 °C min^–1^ to 600 °C and kept for three hours, and then cooled down to room temperature naturally.

### 2.7 Characterization

The X-ray diffraction patterns of the products were collected on a Rigaku-D/max 2500 V X-ray diffractometer with Cu-Kα radiation (*λ* = 1.5418 Å), with an operating voltage and current maintained at 40 kV and 40 mA. Transmission electron microscopic (TEM) images were obtained with a TECNAI G2 high-resolution transmission electron microscope operating at 200 kV. XPS measurements were performed on an ESCALAB-MKII 250 photoelectron spectrometer (VG Co.) with Al Kα X-ray radiation as the X-ray source for excitation.

### 2.8 Catalytic test

20 mg of catalysts were mixed with 20 mg of SiO_2_ powders. The mixture was put in a stainless steel reaction tube. The experiment was carried out under a flow of reactant gas mixture (1% CO, 20% O_2_, balance N_2_) at a rate of 30 mL min^–1^ (SV = 90 000 mL (g^–1^ h^–1^)). The composition of the gas was monitored on-line by gas chromatography (GC 9800).

## 3. Results and discussion

### 3.1 Pd@CeO_2_ core@shell nanospheres

**Fig. 1:**
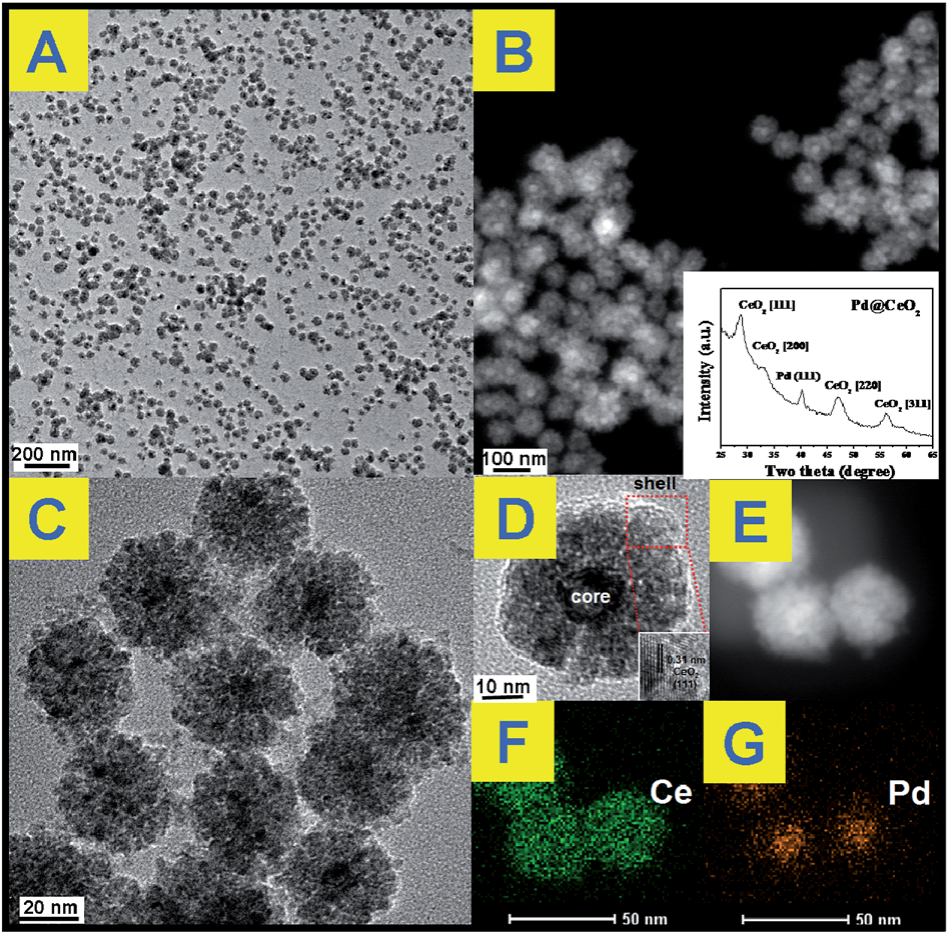
(A), (C) and (D): TEM images; (B) inside; (B) and (E) STEM image; (F) and (G) mapping analysis of the as-obtained Pd@CeO_2_ core@shell nanospheres.

The transmission electron microscope (TEM) images in Fig. 1A show that the as-obtained Pd@CeO_2_ hybrids are uniform and monodisperse sub-45 nm nanospheres. From Fig. 1B–D, it is found that these nanospheres have a core@shell superstructure. In any hybrid nanosphere, the shell is built up by hundreds of tiny CeO_2_ NPs assembling together. The Pd NPs beneath the shell can be distinguished by their deeper contrast from CeO_2_. Every sub-13 nm Pd particle is firmly embedded in the center of 5 nm CeO_2_ nanoparticle aggregation as a single core. It can be seen from the HRTEM images in Fig. 1D that the lattice spacing (0.31 nm) corresponds well with the characteristic (111) planes of fluorite phase CeO_2_. However, the dense CeO_2_ coating makes it hard to observe any clear crystal planes of Pd in the core position, so high-angle annular dark-field scanning transmission electron microscopy (HAADF-STEM) image (Fig. 1E–G) has been used to further analyze the distribution of Pd and Ce components in the nanospheres. It indicates that Ce element spreads everywhere and Pd only exists in the center of the nanospheres, well confirming the Pd@CeO_2_ core@shell nanostructure.

The powder X-ray diffraction (XRD) pattern of the as-obtained sample (Fig. 1B, inset) matches well with those of the standard Pd (JCPDS no. 46-1043) and CeO_2_ (JCPDS no. 34-0394). The X-ray photoelectron spectroscopy (XPS) curves (Fig. S2†) identify that the two peaks at 349.9 eV and 340.4 eV correspond well to the Pd 3d_5/2_ and 3d_3/2_ spin orbit peaks of Pd, respectively, while the peaks at 881.9 eV and 900.2 eV can be assigned to Ce 3d_5/2_ and 3d_3/2_ spin orbit peaks, respectively. The Pd content in the as-obtained Pd@CeO_2_ core@shell nanospheres is 11 wt% determined by elemental analysis using inductively coupled plasma atomic emission spectrometry (ICP-AES). The specific surface area of the as-obtained Pd@CeO_2_ sample calculated from the BET curve (Fig. S3†) reaches about 72.25 m^2^ g^–1^, which is much bigger than that (19.46 m^2^ g^–1^) of our previously reported Pt@CeO_2_ multi-core@shell nanospheres.^40^ This is possibly caused by the smaller size of Pd@CeO_2_ hybrids.

**Fig. 2:**
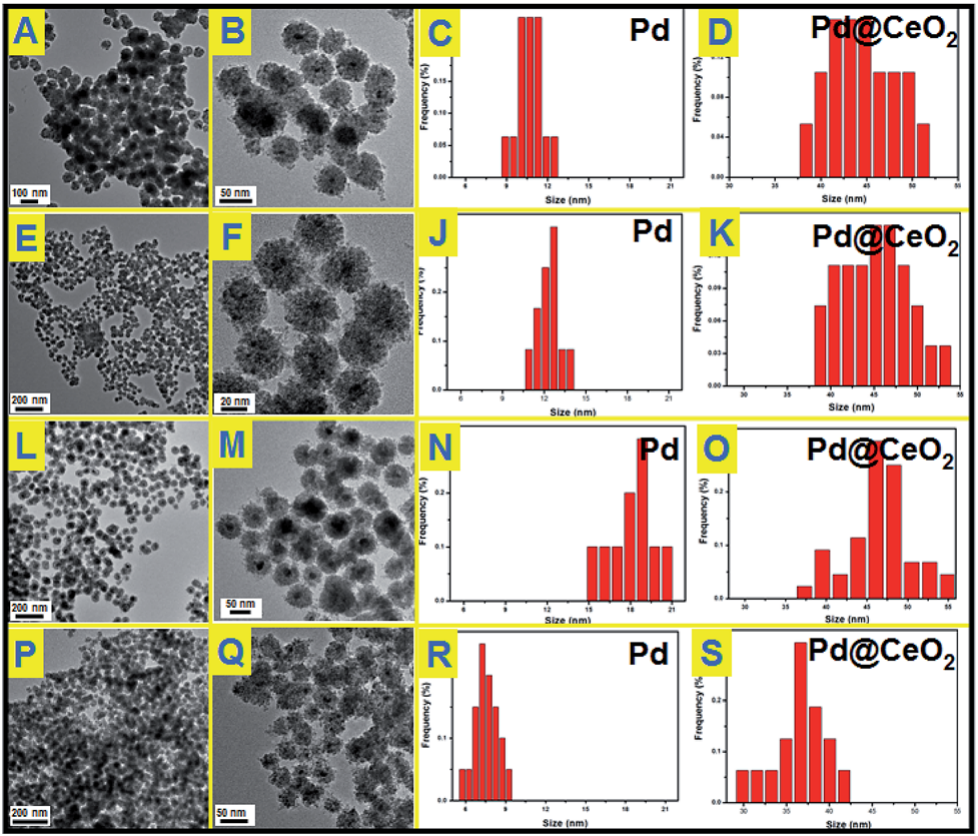
TEM images and particle size distributions of Pd@CeO_2_ core@shell nanospheres obtained by addition of different amounts of KBr in the reaction solution: (A), (B), (C) and (D) 150 mg KBr, 50 μL NH_3_·H_2_O; (E), (F), (J) and (K) 300 mg KBr, 50 μL NH_3_·H_2_O; (L), (M), (N) and (O) 500 mg KBr, 50 μL NH_3_·H_2_O; (P), (Q), (R) and (S) 300 mg KBr, 200 μL NH_3_·H_2_O.

To understand the effects of the feeding amount of KBr and NH_3_·H_2_O on the formation of Pd@CeO_2_ core@shell nanospheres, a series of control experiments have been designed to get a deeper insight into how the core@shell nanostructure evolved. We first studied the usage amount of KBr on the structural evolution by TEM characterization. As shown in Fig. S4,† no uniform hybrid nanostructure could be found in the absence of Br^–^. The corresponding EDX analysis (Fig. S5†) can identify that these irregular NPs are disorderly mixed Pd and CeO_2_ components. After adding 50 mg of KBr, each Pd nanoparticle began to be surrounded by hundreds of small CeO_2_ NPs to form a core@shell-like structures, but they aggregated seriously (Fig. S6†), and many un-coated Pd NPs could be easily found from the TEM images. With increasing the KBr amount to 150 mg, the core@shell spherical nanostructure became more clear, uniform and monodisperse (Fig. 2A and B). The Pd NPs grow from 11 nm to 13 nm to 17 nm on average while increasing the KBr amount from 150 mg to 300 mg to 500 mg as shown in Fig. 2C–F. If the NH_3_·H_2_O amount was increased to 200 μL in the presence of 300 mg of KBr, we could still obtain core@shell structured nanospheres as shown in Fig. 2P and Q, however both of the core@shell nanospheres and the Pd cores became much smaller. It means the increased alkalinity of the solution accelerated the aggregation of CeO_2_ NPs to give the Pd cores a timely stronger protection, resulting in the smaller size of Pd@CeO_2_ core@shell nanospheres as well. However, if we use NaOH to replace NH_3_·H_2_O, the product is an aggregation composed by numerous small NPs mixed together, forming the disordered Pd–CeO_2_ hybrids as shown in Fig. S7.†

The used amounts of KBr and NH_3_·H_2_O as well as the final sizes of the Pd core and the nanosphere have been summarized in Table 1. The results show that the sizes of Pd NPs are strongly dependent on the feeding amount of KBr as well as NH_3_·H_2_O. As reported, Br^–^ ions can retard the reduction of Pd^2+^ ions *via* the formation of [PdBr_4_]^2–^, because the overall stability constant of [PdBr_4_]^2–^ is nearly 104 times higher than that of [PdCl_4_]^2–^ while [Br^–^]/[Cl^–^] = 7.5.^40^–^42^ According to the Nernst equation, the potential of Pd^2+^ is greatly reduced due to the formation of a more stable complex [PdBr_4_]^2–^.^43^ Obviously stable coordination of Pd^2+^ ions does not favor the independent nucleation of a Pd nanoparticle but its growth. This could give a good explanation for the phenomenon that the Pd NPs grew bigger and bigger while continuously increasing the used amount of KBr from 0 to 500 mg. However the self-assembly behavior of Pd and CeO_2_ remains still unclear, which is most likely driven by thermodynamics. Here we have tried to propose the salting-out effect to clarify this process.

**Table 1:** The usage amounts of KBr, NH_3_·H_2_O and the size of products

KBr (mg)	NH_3_·H_2_O (μL)	Nanosphere (nm)	Pd (nm)	CeO_2_ (nm)
0	50	—	<9	5
150	50	43	11	5
300	50	44	13	5
500	50	46	17	5
300	200	36	8	5

Generally, introducing a mass of inorganic salts to the mother aqueous solution will make the original solute supersaturated and thus being separated by crystallization. These salts can somehow weaken the polarity of the solute, resulting in reduced solubility in water. Comparatively the difference in our case is that the solutes were Pd and CeO_2_ NPs. So once KBr was introduced into the solution, these surface polarity-weakened Pd and CeO_2_ NPs in water have to spontaneously self-assemble into more stable and ordered structures due to highly decreased colloidal stability. Consequently, a large amount of Pd@CeO_2_ core@shell nanosphere deposits was observed and easily collected from the reaction solution after standing for about 30 min. As shown in Fig. S8,† after purification, these black deposits could be well re-distributed in water to form a very stable colloid until KBr was added again. The deposition–purification process can be successfully repeated for cycles by addition of KBr into the colloids.

### 3.2 Au@CeO_2_ and Pt@CeO_2_ core@shell nanostructures

**Fig. 3:**
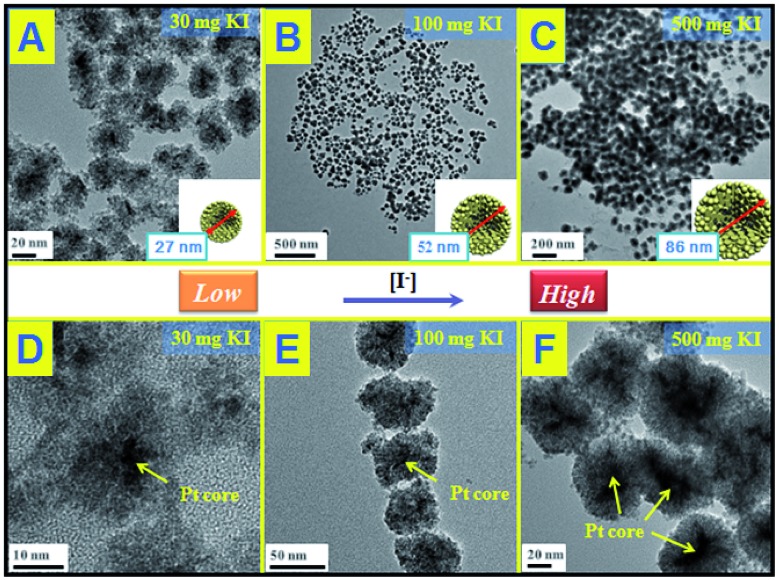
TEM images of Pt@CeO_2_ multi-core@shell nanospheres prepared by KI assistance; (A) and (D): 30 mg; (B) and (E): 100 mg; (C) and (F): 500 mg.

Other kinds of halogen ions have been also studied instead of Br^–^. As shown in Fig. S9 and S10,† it is found that neither Cl^–^ nor I^–^ works to induce the self-assembly behavior between Pd and CeO_2_, demonstrating that the inducing effect of Br^–^ is specific for the Pd–CeO_2_ system. The growth mechanism of the core@shell nanostructures could be also understood as the salting-out effect as mentioned above (as shown in Scheme 1). The precipitation of two components is accompanied by a self-assembly process to form the more stable core@shell nanoparticles. Following this principle, we successfully use KCl and KI to induce the formation of Au@CeO_2_ and Pt@CeO_2_ nanostructures, respectively. As shown in Fig. S11 and S12,† under the similar reaction conditions (300 mg of KBr), Br^–^ couldn't induce the formation of either Au@CeO_2_ or Pt@CeO_2_ nanospheres. Unexpectedly, KCl specifically works towards Au–CeO_2_, while KI towards Pt–CeO_2_. For the KCl–Au–CeO_2_ system, until 300 mg KCl were added in the reaction solution, a core@shell nanostructure similar to Pd@CeO_2_ could be also observed. (Fig. S13†). While for the KI–Pt–CeO_2_ system as shown in Fig. 3, just a small amount of KI could induce the formation of uniform small Pt@CeO_2_ multi-core@shell nanospheres. The higher the concentration of KI in the reaction solution, the bigger the hybrid nanospheres. 30, 100 and 500 mg KI could assist the fabrication of 27, 52 and 86 nm Pt@CeO_2_ nanospheres, respectively.

### 3.3 Pd@CeO_2_ supported on γ-Al_2_O_3_

**Fig. 4:**
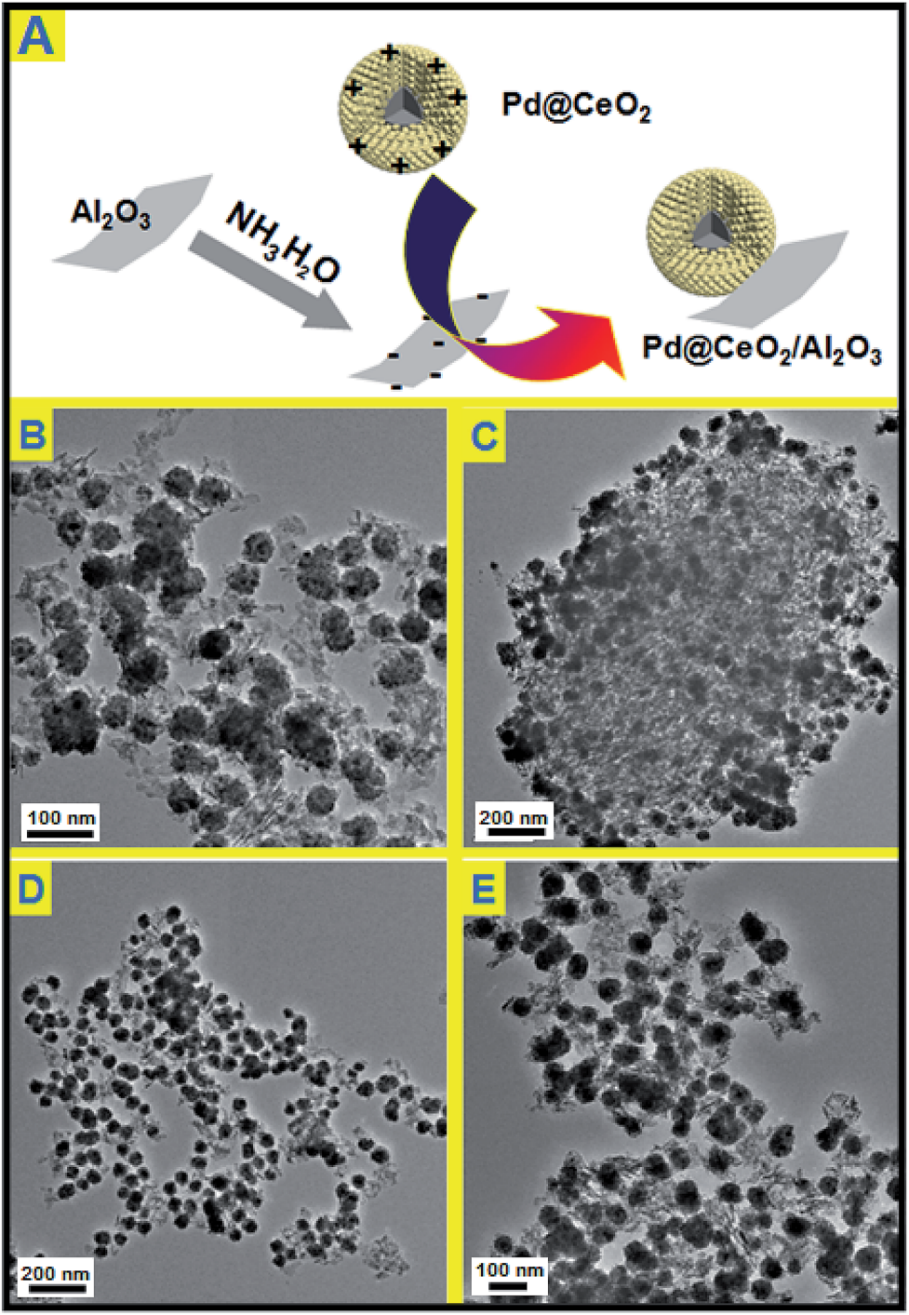
(A) Schematic representation of the self-assembly of Pd@CeO_2_ and commercial γ-Al_2_O_3_; (B) TEM images of 11 nm-Pd@CeO_2_/Al_2_O_3_; (C) 13 nm-Pd@CeO_2_/Al_2_O_3_; (D) 17 nm-Pd@CeO_2_/Al_2_O_3_; (E) 8 nm-Pd@CeO_2_/Al_2_O_3_.

Electrostatic attraction is a universal force that exists between oppositely charged components. It is noticed the as-obtained Pd@CeO_2_ core@shell nanospheres are positively charged (zeta potential: +25.3). So in this consideration, negatively charged γ-Al_2_O_3_ was supposed to be able to support the Pd@CeO_2_ core@shell nanospheres. The commercial γ-Al_2_O_3_ is neutral, however, their surface can carry negative charges (zeta potential: –15.5) if refluxed in aqueous NH_3_·H_2_O. As expected once the Pd@CeO_2_ colloids were added into the solution of the negative γ-Al_2_O_3_, the black precipitation appeared and could be easily collected at the bottom of the flask. The TEM images (Fig. 4) show that the as-prepared Pd@CeO_2_ core@shell samples with different Pd particle sizes have been successfully loaded onto the needle-like commercial γ-Al_2_O_3_ supports uniformly and kept their original monodispersity. No scattered or agglomerated ones are found. This could be attributed to the strong electrostatic attraction between CeO_2_ and γ-Al_2_O_3_ and the electrostatic repulsion between Pd@CeO_2_ nanospheres themselves. Compared with the previous report by Gorte's group, such pure inorganic loading strategy greatly simplifies the synthetic steps.^11^,^33^–^36^

### 3.4 Thermal stability

**Fig. 5:**
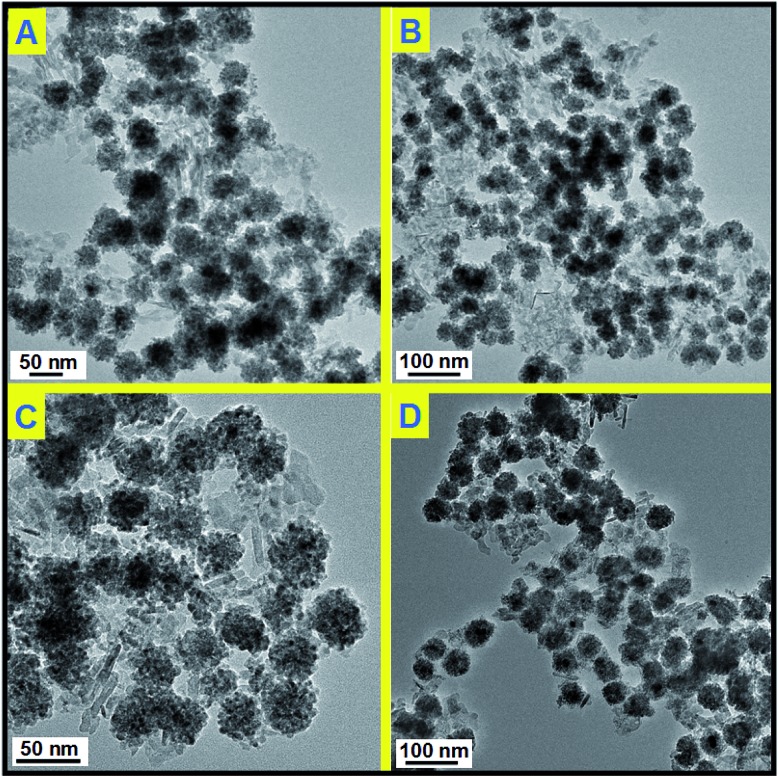
TEM images of Pd@CeO_2_/Al_2_O_3_ products after heating at 600 °C for 3 hours; (A) 11 nm; (B) 13 nm; (C) 17 nm; (D) 8 nm.

Before catalytic tests, the thermal stability of Pd@CeO_2_/Al_2_O_3_ was first examined by calcination at 600 °C for three hours in air. By comparing the TEM images (Fig. 5), it is found that there is no change in size, shape and structure, and no agglomeration happened in the cases of all the four Pd@CeO_2_ samples. Their excellent thermal ability could be attributed to the strong protection of the CeO_2_ shell and the support of γ-Al_2_O_3_.

### 3.5 Catalytic CO oxidation

**Fig. 6:**
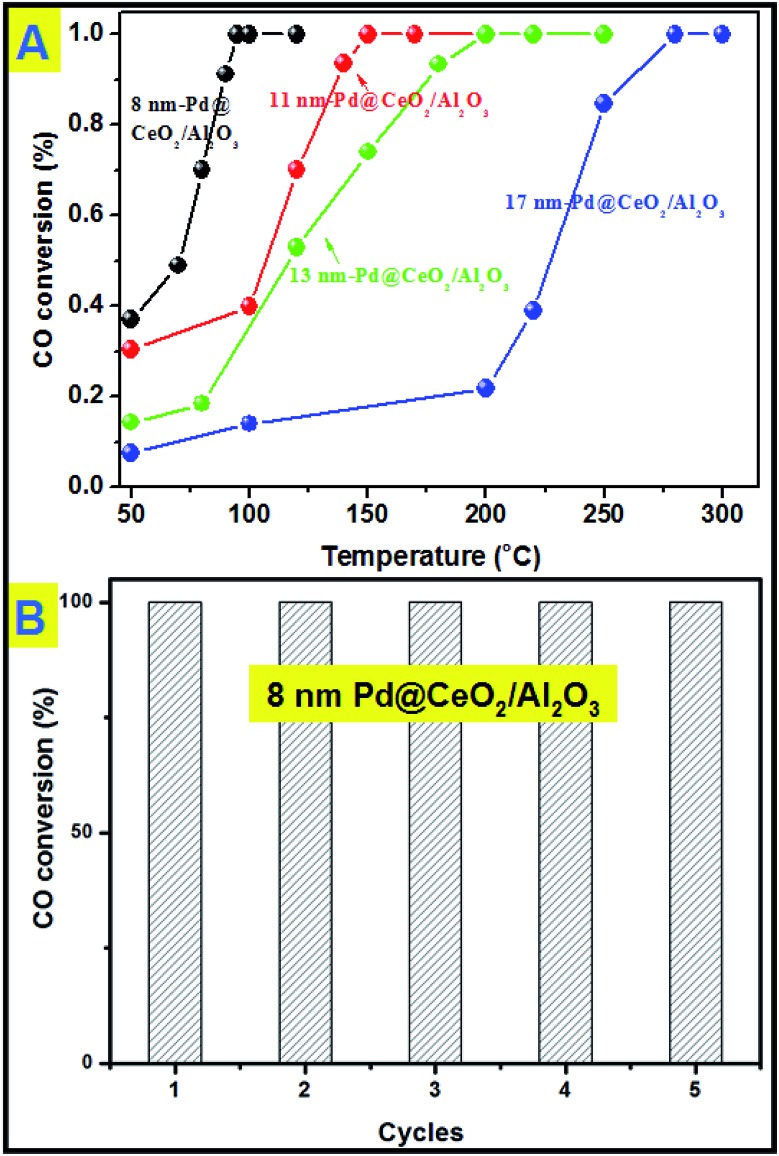
(A) catalytic CO conversion of Pd@CeO_2_/Al_2_O_3_ with different Pd core sizes; (B) cycling test of 8 nm-Pd@CeO_2_/Al_2_O_3_.

All the samples have been thermally treated in advance at 600 °C for three hours before every catalytic test. Then catalytic CO oxidation into CO_2_ has been chosen as the model reaction to study the catalytic activity and stability of the as-obtained Pd@CeO_2_ and Pd@CeO_2_/Al_2_O_3_ samples. The evaluation of catalytic activity was performed in a fixed-bed reactor coupled online with a gas chromatograph (see the catalytic activity measurement for CO oxidation in the Experimental section). Fig. 6A shows the typical conversion ratio of CO as a function of reaction temperature over five catalysts under the conditions that the feed gas containing 1 vol% CO and 99 vol% air was allowed to pass through the reactor at a total flow rate of 50 mL min^–1^, corresponding to a gas hourly space velocity (GHSV) of 15 000 mL h^–1^ g^–1^ cat. CO conversion curves in Fig. 6 show that the Pd@CeO_2_/Al_2_O_3_ samples exhibited a size-dependent catalytic performance. The *T*_100_ (100% conversion temperature) of the four samples follows such a sequence: 8 nm-Pd@CeO_2_/Al_2_O_3_ (95 °C) < 11 nm-Pd@CeO_2_/Al_2_O_3_ (145 °C) < 13 nm-Pd@CeO_2_/Al_2_O_3_ (250 °C) < 17 nm-Pd@CeO_2_/Al_2_O_3_ (280 °C). The 8 nm-Pd@CeO_2_/Al_3_O_4_ has a lower *T*_100_ of about 95 °C compared with the previous report by Gorte's group (about 110 °C),^29^ which is even much better than the currently reported Pt@CeO_2_ (135 °C),^36^ Au@CeO_2_ (155 °C)^13^ and Pt–CeO_2_ (140 °C)^44^ systems.

Generally, smaller Pd NPs are more active in catalytic CO oxidation. However, compared with the 2 nm-Pd@CeO_2_ core@shell nanospheres,^29^ the as-obtained 8 nm-Pd@CeO_2_/Al_2_O_3_ show better performance. This should be attributed to our clean synthesis route that favors the formation of a more strongly coupled interface between the Pd and CeO_2_ components, resulting in its excellent catalytic performance. Besides, if we compare the catalytic performance of the Pd–CeO_2_ sample obtained without addition of KBr with that of 8 nm-Pd@CeO_2_ (Fig. S14†) and with that of 8 nm-Pd@CeO_2_/Al_2_O_3_, it is clearly found that an ordered self-assembled core@shell structure and Al_2_O_3_ support are both beneficial for improving the activity of Pd catalysts.

Next, a cycling test was done to study the stability of 8 nm-Pd@CeO_2_/Al_2_O_3_, and before every cycle it was calcined in advance at 600 °C in air. As shown in Fig. 6B, after five successful cycles 8 nm-Pd@CeO_2_/Al_2_O_3_ still maintained 100% conversion rate of CO into CO_2_ at the testing temperature of 100 °C. In the TEM image taken after the cycling test (Fig. S15†) it is seen that 8 nm-Pd@CeO_2_/Al_2_O_3_ kept their original core@shell nanostructure well, indicating its excellent catalytic performance under long-term high-temperature catalytic conditions.

**Fig. 7:**
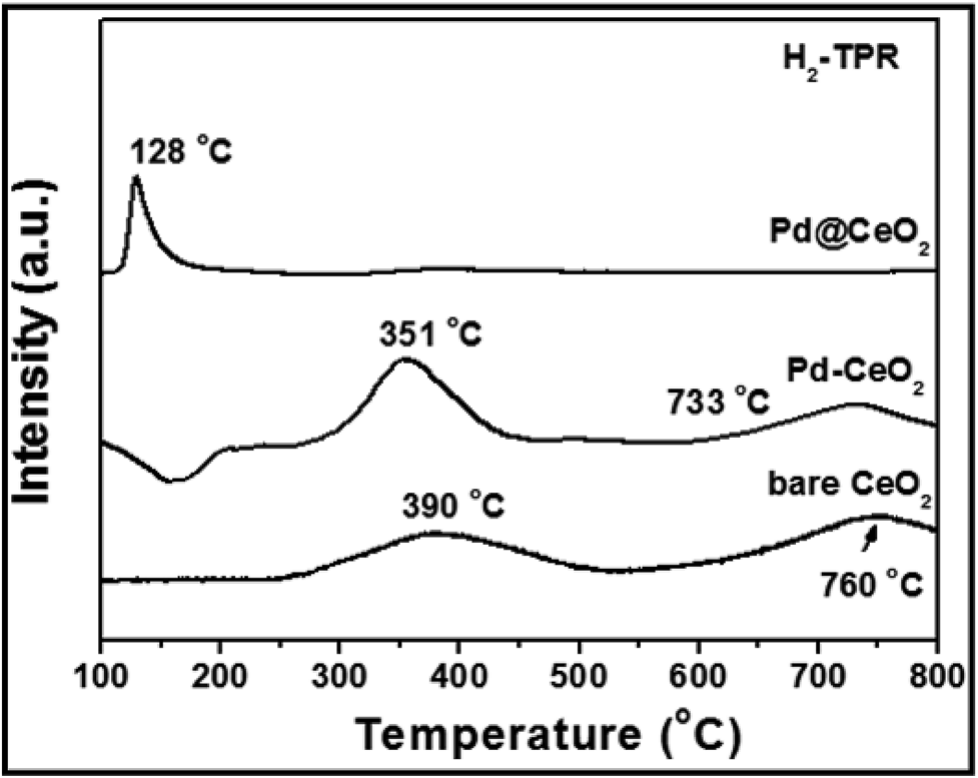
H_2_-TPR spectra of 8 nm-Pd@CeO_2_, Pd–CeO_2_ and bare CeO_2_ samples.

Research has been focused on the reactivity occurring at the interface of noble metal/ceria hybrids.^45^ In order to investigate the importance of maximizing the metal–support interaction by the core@shell approach, H_2_-TPR (H_2_-temperature programmed reduction) test has been done and is shown in Fig. 7. Two broad peaks observed at 390 and 760 °C for bare CeO_2_ can be attributed to the reduction of surface capping oxygen and bulk oxygen of CeO_2_, respectively.^46^ While, in the curve of Pd–CeO_2_ (prepared without addition of KBr), the two peaks have been reduced to 351 and 733 °C from 390 and 760 °C, respectively. However for 8 nm-Pd@CeO_2_, an intense and sharp peak could be clearly observed at 128 °C. The greatly reduced temperature indicates its higher oxygen delivery capacity which is strongly related to the ultra-high catalytic performance of the core@shell nanostructure.

## 4. Conclusion

In conclusion, we have successfully developed a novel and low-cost auto-redox strategy to construct the Pd@CeO_2_ core@shell nanostructures with tunable Pd core sizes. The reaction is triggered between Ce(OH)_3_ and Na_2_PdCl_4_ in air without addition of any reducing agents or surfactants except for using KBr. The as-obtained Pd@CeO_2_ nanospheres are uniform and completely built up by spontaneously self-assembled naked Pd and CeO_2_ NPs. KBr here is reasonably supposed to play two key roles in determining the growth of Pd NPs and the final self-assembled hybrid spherical structures. First, Br^–^ ions retarded the reduction of Pd^2+^ ions *via* the formation of the more stable complex of [PdBr_4_]^2–^ so as to be able to tune the size of Pd cores. Second, due to the “salting-out effect” in water, it greatly decreases the colloidal stability, and hence the surface polarity-weakened Pd and CeO_2_ NPs have to spontaneously self-assemble into more stable and ordered structures. Besides interestingly, the similar “salting-out effect” strategy works well in the formation of other noble metal@CeO_2_ hybrids. It shows specific inducing effects that Cl^–^ is specific towards the Au@CeO_2_ system, Br^–^ towards Pd@CeO_2_, and I^–^ towards Pt@CeO_2_.

With the help of electrostatic interactions, these positively charged Pt@CeO_2_ samples can be uniformly and strongly supported on the negatively charged Al_2_O_3_. Thanks to their ordered self-assembled core@shell structure and Al_2_O_3_ support, these Pd catalysts exhibited excellent activity and stability in catalytic CO oxidation. Among different-sized Pd samples, the as-obtained 8 nm-Pd@CeO_2_/Al_2_O_3_ shows the best performance, which can catalyze 100% CO conversion into CO_2_ at a relatively low temperature of 95 °C compared with those of the previously reported CeO_2_-encapsulated Pd samples. It is believed that such a “salting-out effect” strategy is of great significance in the synthesis of self-assembled inorganic NPs and design of highly active and stable catalysts in real-world applications.

## Supplementary Material

Supplementary information
