# Supplementary material for: γ-Al2O3 supported Pd@CeO2 core@shell nanospheres: salting-out assisted growth and self-assembly, and their catalytic performance in CO oxidation
Source: Chem Sci. 2015 Feb 27;6(5):2877–84. doi: 10.1039/c4sc03854a (PMC5729413; doi:10.1039/c4sc03854a)
Supplement: Supplementary file 1 [file SC-006-C4SC03854A-s001.pdf]

Electronic Supplementary Material (ESI) for Chemical Science.
This journal is © The Royal Society of Chemistry 2015

Supporting Information

γ-Al_2O_3 Supported Pd@CeO_2 Core@Shell Nanospheres: Salting-out Assisted Growth and Self-assembly, and Their Catalytic Performance on CO Oxidation

Xiao Wang, Dapeng Liu, Junqi Li, Jiangman Zhen, Fan Wang, and Hongjie Zhang**

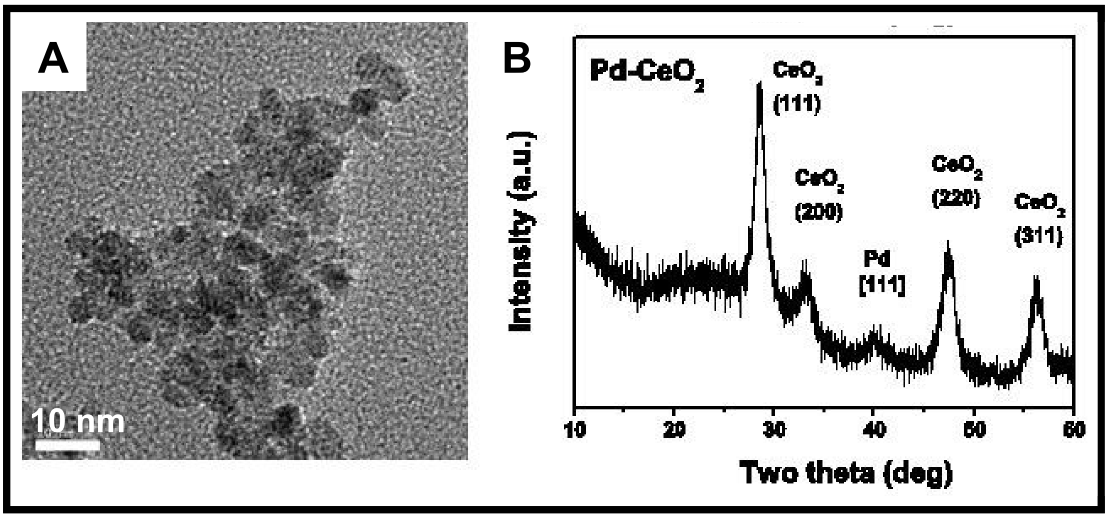

Figure S1. TEM (A) and XRD pattern (B) of the Pd-CeO_2 hybrids which are prepared according to our previously reported Pt@CeO_2 system.

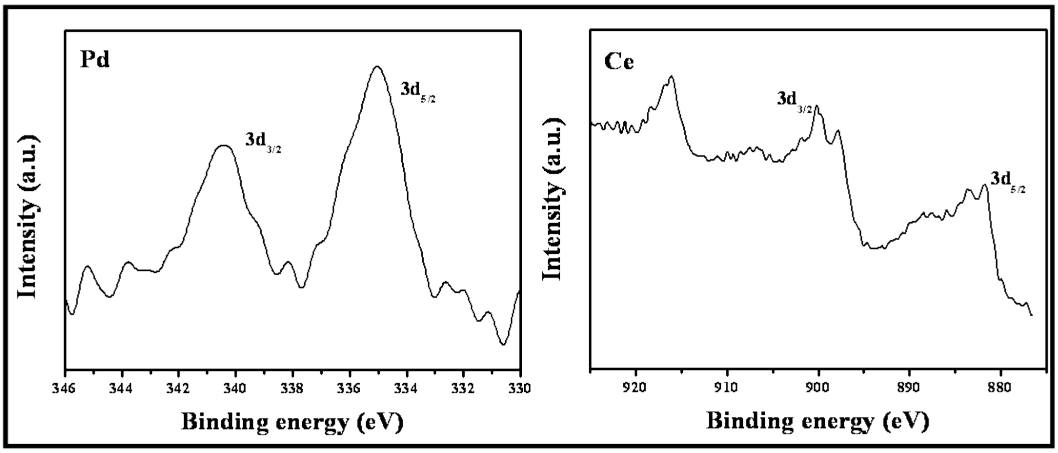

Figure S2. XPS analysis of Pd and Ce in Pd@CeO_2.

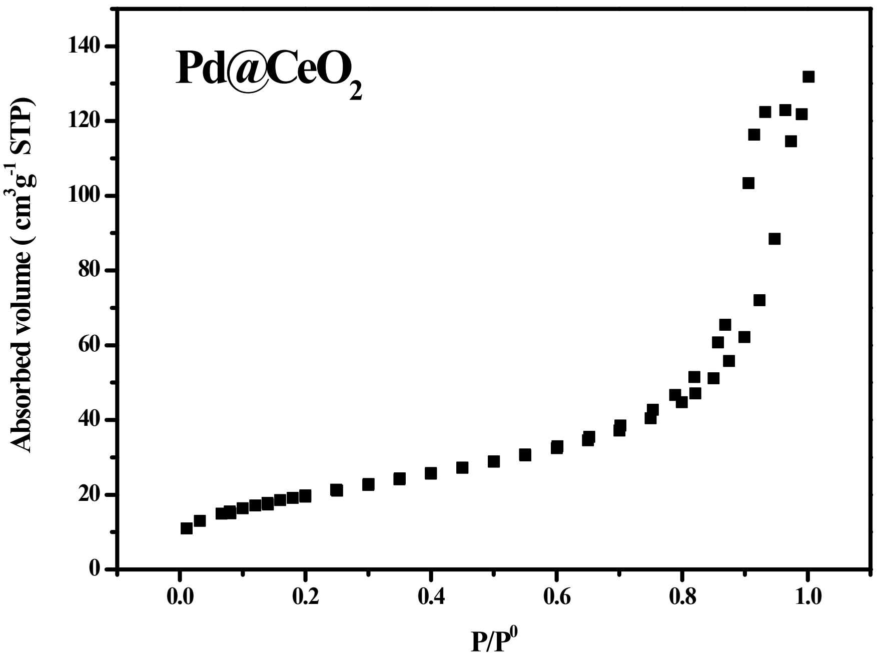

Figure S3. The BET curve of 13 nm-Pd@CeO_2.

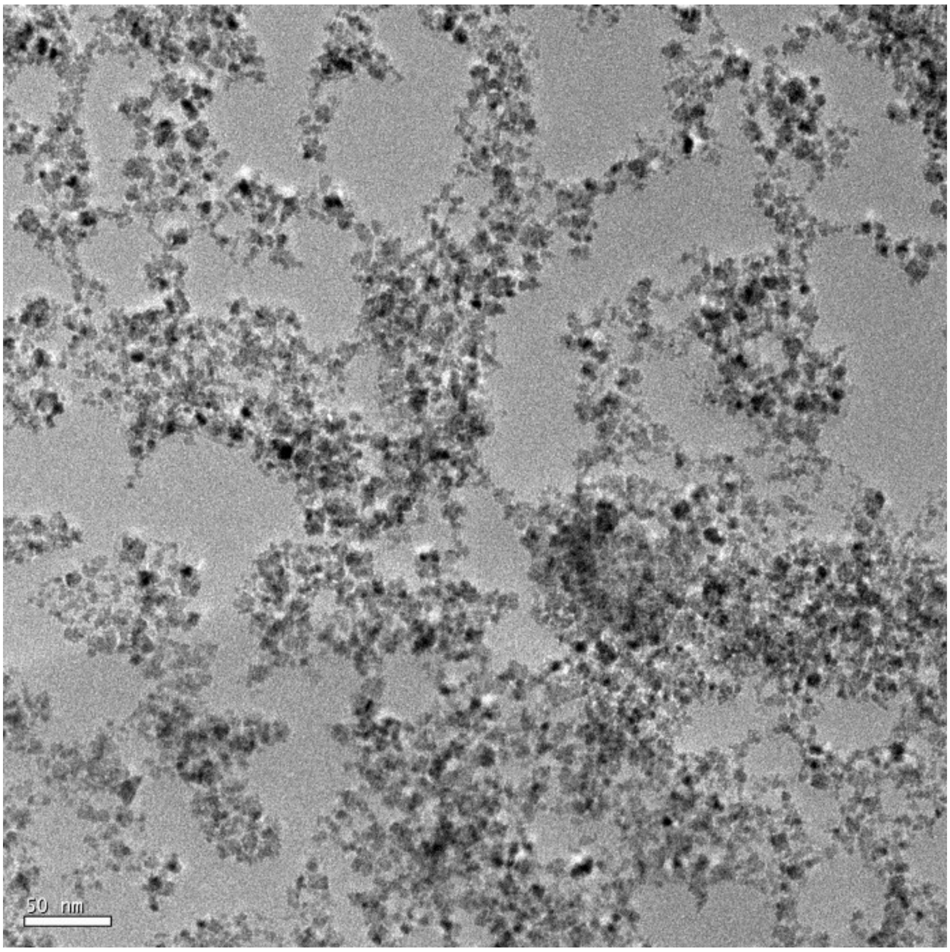

Figure S4. TEM images of Pd-CeO_2 prepared without using KBr.

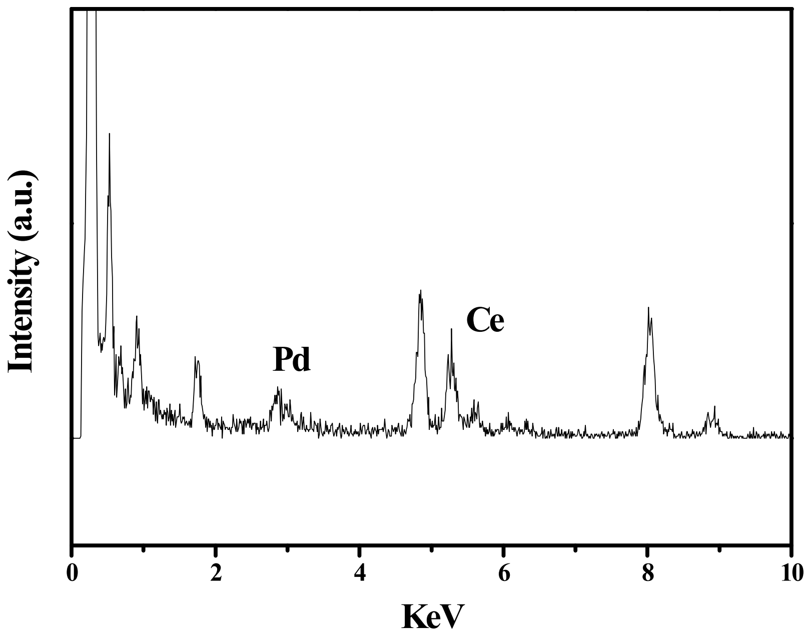

Figure S5. EDX curve of Pd-CeO_2 sample prepared without using KBr.

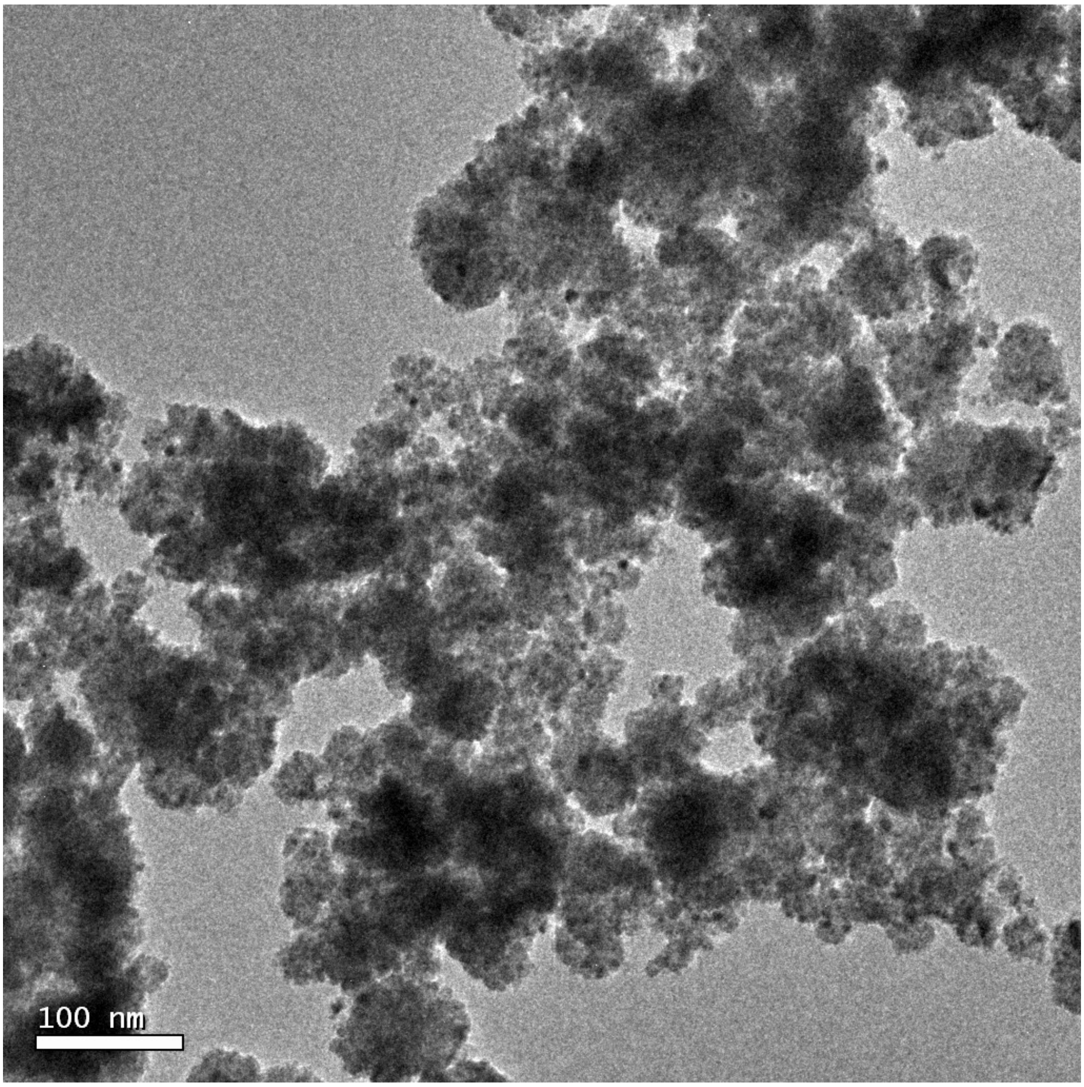

Figure S6. TEM images of Pd-CeO_2 synthesized by addition of 50 mg KBr.

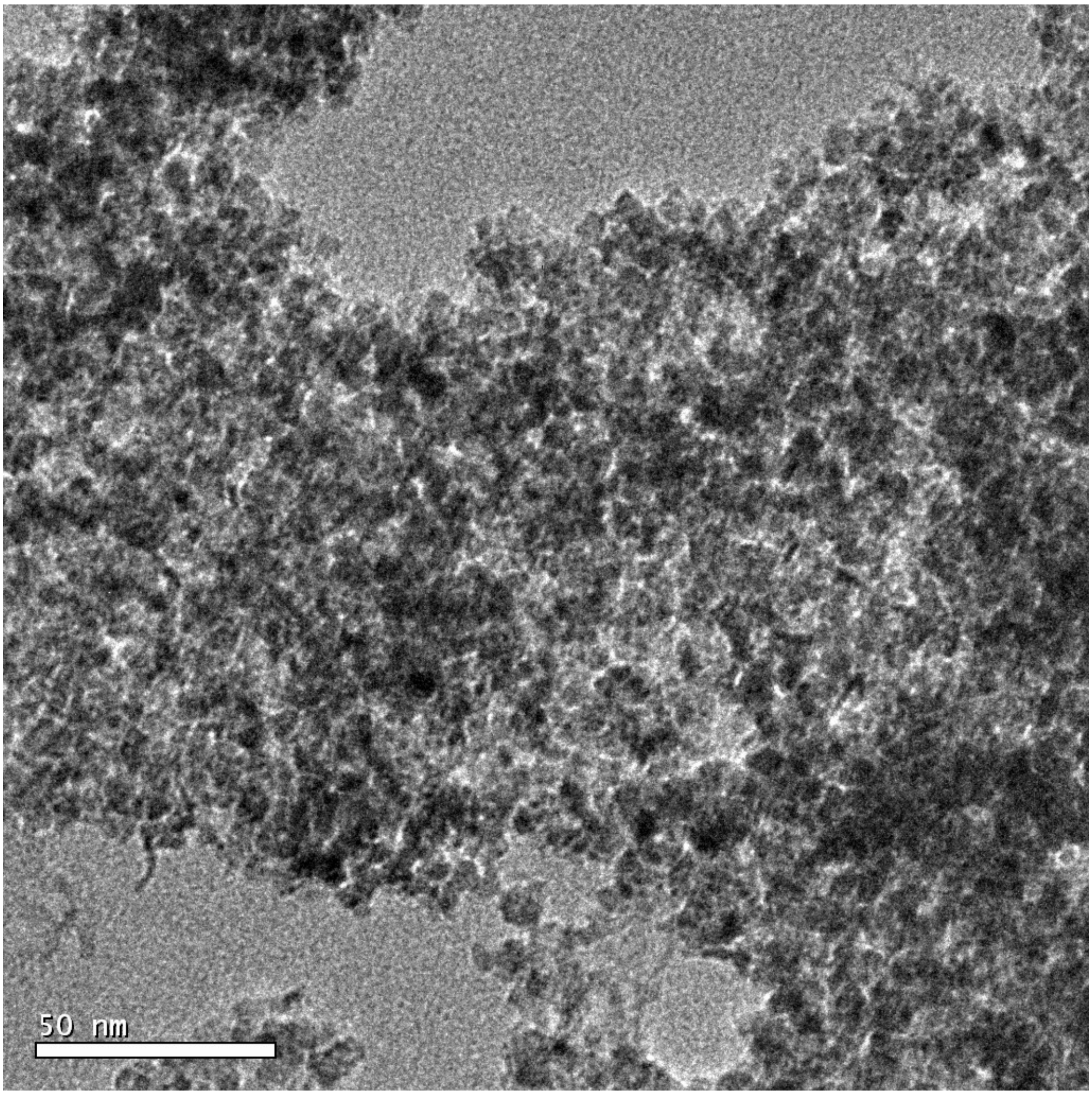

Figure S7. TEM image of Pd@CeO_2 prepared by using NaOH instead of NH_3•H_2O.

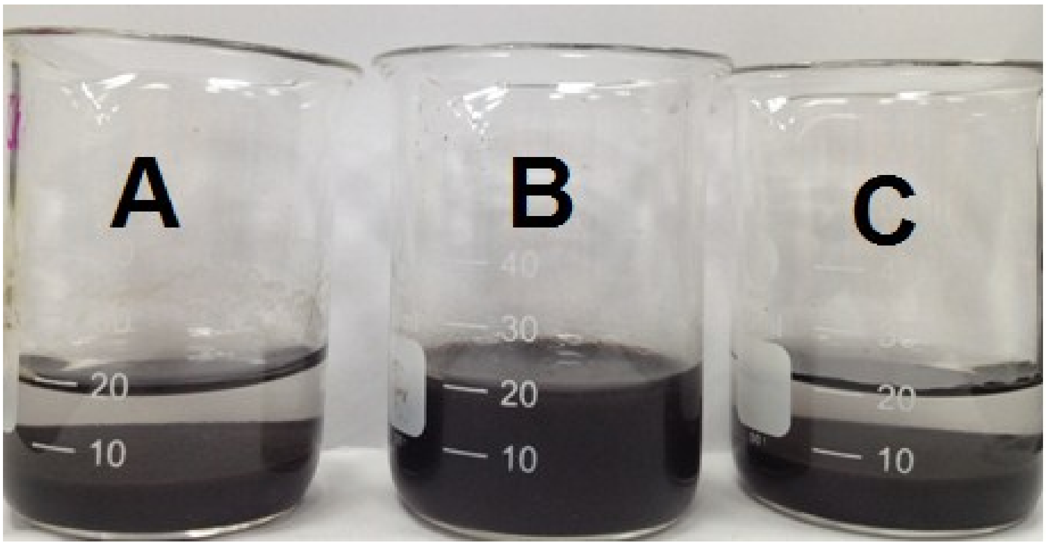

Figure S8. Photos of (A) the freshly prepared Pd@CeO_2 sample after standing for 30 min; (B) redistribution of purified Pd@CeO_2 in water after standing for 30 min in the absence of KBr; (C) redistribution of purified Pd@CeO_2 in 2 M KBr solution after standing for 30 min.

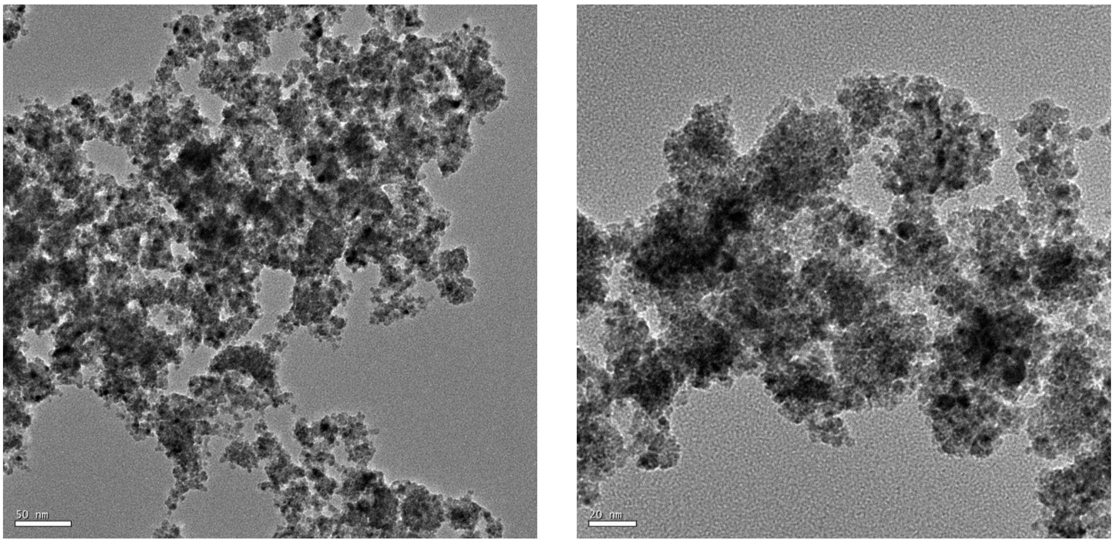

Figure S9. TEM images of Pd@CeO_2 prepared by using KCl (300 mg) instead of KBr (300 mg).

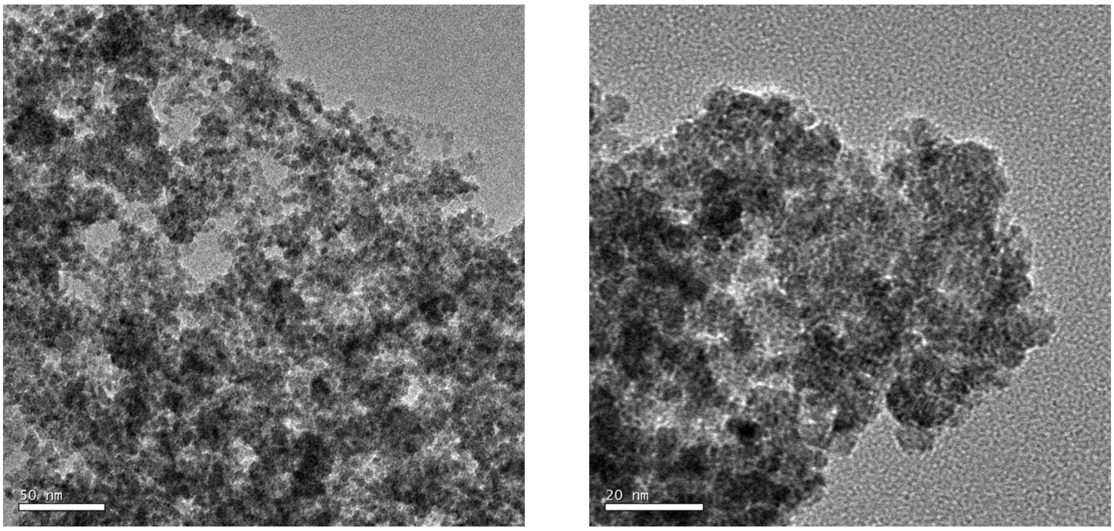

Figure S9. TEM images of the sample prepared by using KI (300 mg) instead of KBr (300 mg).

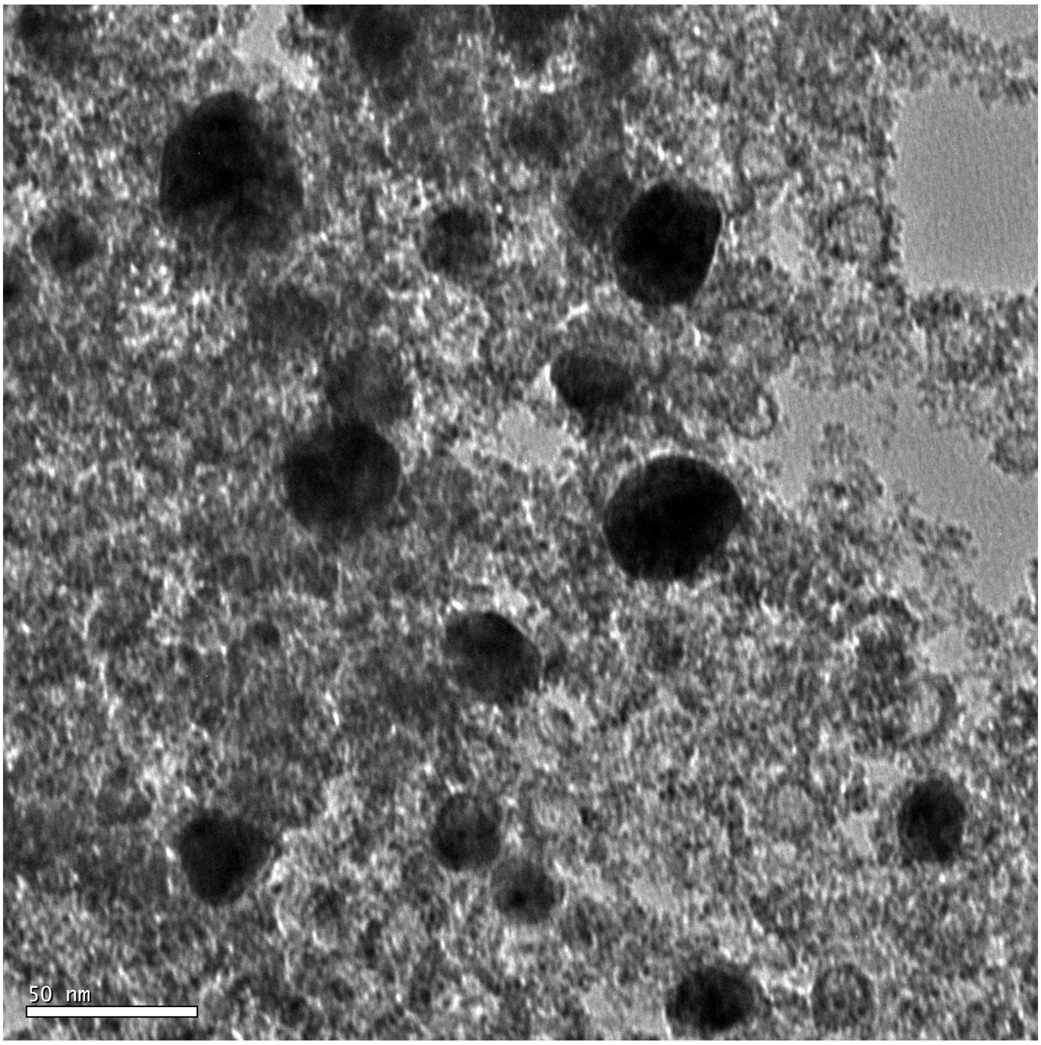

Figure S11. TEM image of Au-CeO_2 hybrid prepared by using KBr (300 mg).

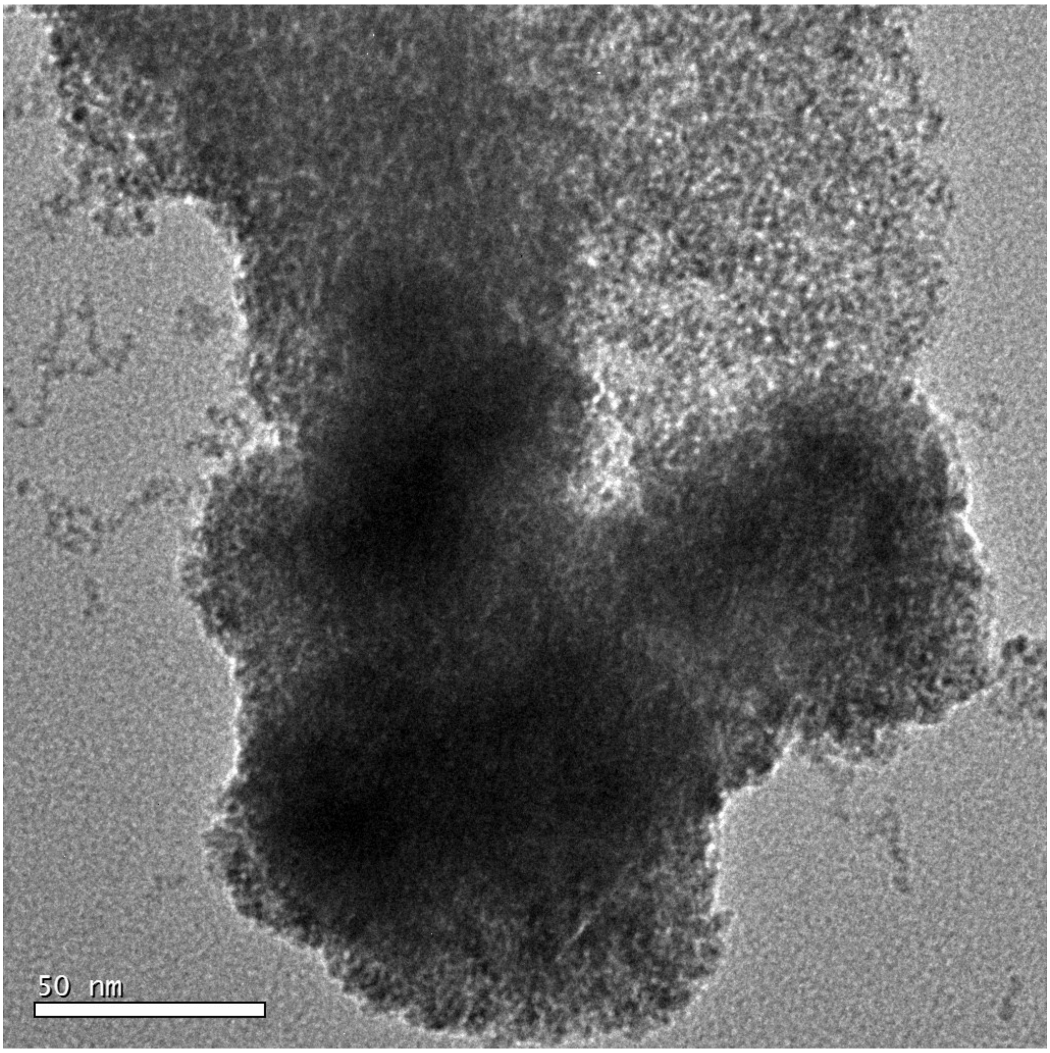

Figure S12. TEM image of Pt-CeO_2 hybrid prepared by using KBr (300 mg).

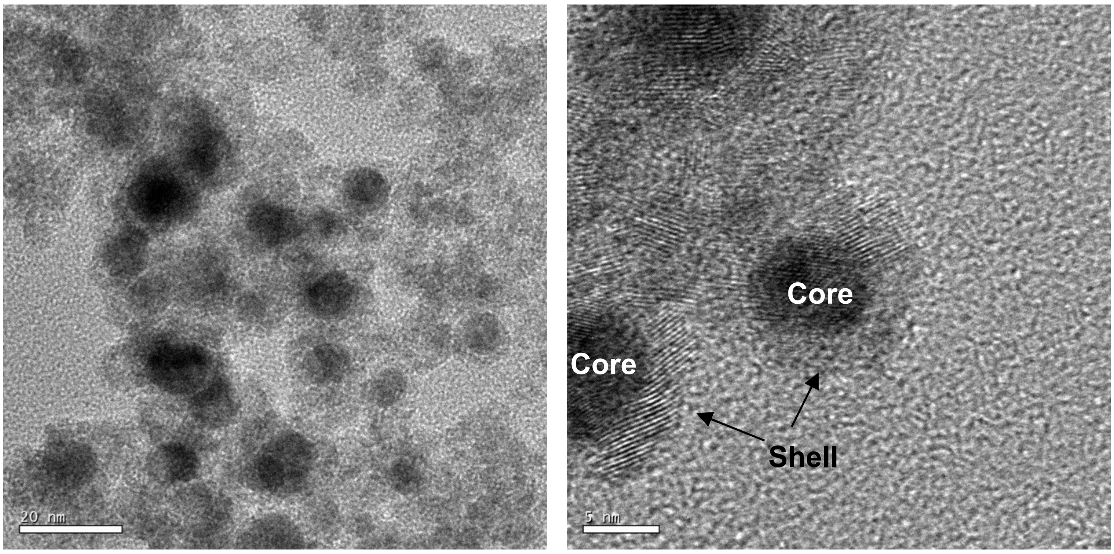

Figure S13. TEM images of Au@CeO_2 core@shell nanostructures prepared by using KCl (300 mg).

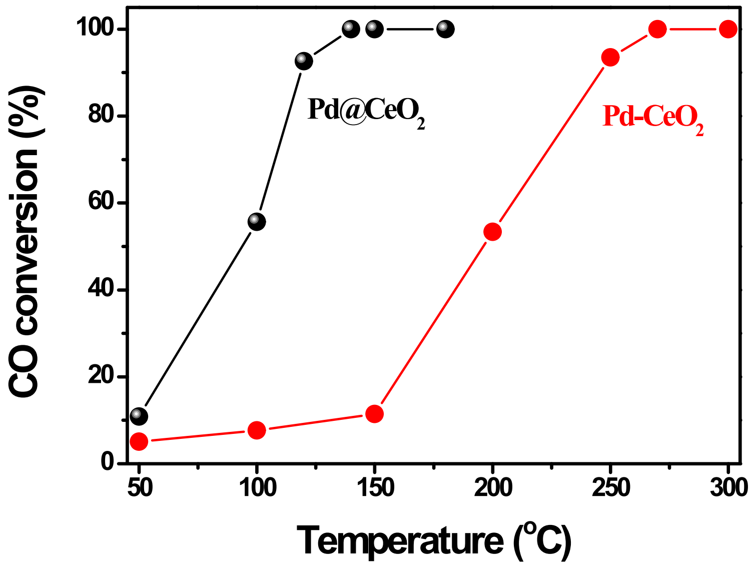

Figure S14. Catalytic CO oxidation curves of 8nm-Pd@CeO_2 and simply loaded Pd-CeO_2 samples after heat treatment.

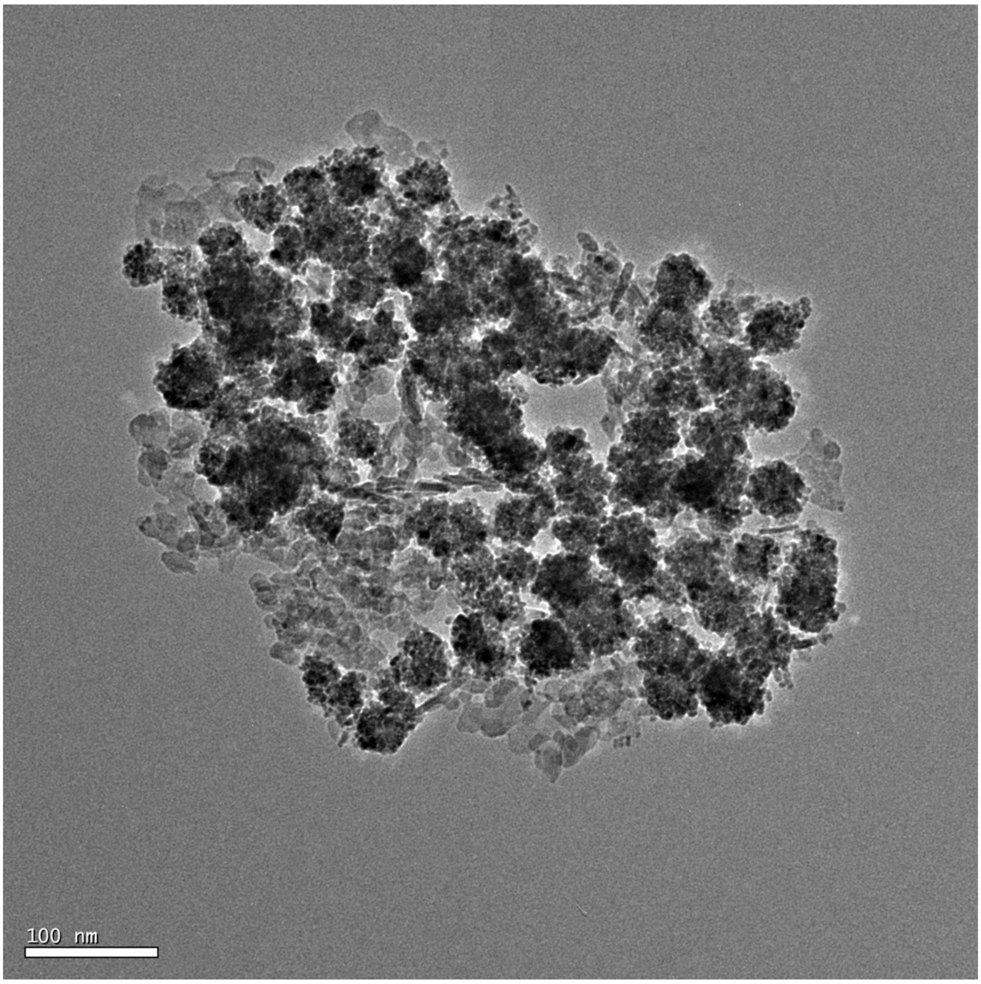

Figure S15. TEM images of 8 nm-Pd@CeO_2/Al_2O_3 after the cycling test.
